# Constraints on Non-Newtonian Gravity From the Experiment on Neutron Quantum States in the Earth’s Gravitational Field

**DOI:** 10.6028/jres.110.037

**Published:** 2005-06-01

**Authors:** V. V. Nesvizhevsky, K. V. Protasov

**Affiliations:** Institut Laue Langevin, Grenoble, France; Laboratoire de Physique Subatomique et de Cosmologie, Grenoble, France

**Keywords:** neutron quantum states, non-Newtonian gravity, supplementary dimensions

## Abstract

An upper limit to non-Newtonian attractive forces is obtained from the measurement of quantum states of neutrons in the Earth’s gravitational field. This limit improves the existing constraints in the nanometer range.

## 1. Introduction

According to the predictions of unified gauge theories, supersymmetry, supergravity, and string theory, there would exist a number of light and massless particles [1]. An exchange of such particles between two bodies gives rise to an additional force. Additional fundamental forces at short distances were intensively studied following the hypothesis about “large” supplementary spatial dimensions proposed in [2]. For a review of theoretical works and recent experimental results, see [3,4]. This hypothesis could be verified using neutrons because the absence of an electric charge allows one to strongly suppress the false electromagnetic effects [5]. It was noticed in [6] that the measurement of the neutron quantum states in the Earth’s gravitational field [7] is sensitive to such extra forces in the sub-micrometer range. In the case of three extra dimensions, the characteristic range is just in the nanometer domain [2,5] which is accessible in this experiment. The first attempt to establish a model-dependent boundary in the range from 1 µm to 10 µm, was presented in Ref. [8]. In this contribution, we estimate an upper limit on an additional attractive short-range force, which could be established from this experiment in a model-independent way [9].

An effective gravitational interaction in presence of an additional Yukawa-type force is parametrized as:

$$V_{\mathrm{eff}}\;(r)=G\frac{m_{1}m_{2}}{r}\left(1+\alpha_{G}e^{-r/\lambda }\right)$$ (1)

Here, *G* is the Newtonian gravitational constant, *m*_1_ and *m*_2_ are interacting masses, *r* their relative distance, *α_G_* and *λ* are strength and characteristic range of this interaction.

The experiment [7] consists in the measurement of the neutron flux through a slit between a horizontal mirror on bottom and a scatterer/absorber on top as a function of the slit size. The motion of neutrons in this system over the vertical axis *z* could be considered as a one-dimensional problem for which the mirror provides an infinitely high potential. The interaction between neutrons and the Earth is described by the first term in Eq. (1) and can be approximated by the usual linear potential (*r* = *R* + *z*):

V(z)=mgz (2)

with *g* = *GM*/*R*^2^, *R* being the Earth’s radius, *M* its mass, *m* the neutron mass.

The second term in Eq. (1) introduces an additional interaction. Due to the short range of this interaction, its main contribution is provided by the interaction of neutrons with a thin surface layer of the mirror and the scatterer. An additional potential of this interaction is given by:

$$V\prime (z)=-U_{0}e^{-z/\lambda }$$ (3)

with *U*_0_ = 2π*Gα_G_mρ*_m_*λ*^2^, *ρ*_m_ being mirror’s density.

## 2. Attractive Interaction

The simplest upper limit on the strength of an additional interaction follows from the condition that this additional interaction does not create itself any bound state. For an exponential attractive (*α_G_* > 0) potential [Eq. (2)] this means that:

$$\frac{U_{0}m\lambda^{2}}{h^{2}}<0.72.$$ (4)

This condition gives a boundary for an additional potential strength:

$$\alpha_{G}=0.72\frac{2}{\pi }\frac{\rho }{\rho_{m}}\frac{h}{mg\lambda^{2}}\frac{h}{m\lambda }\frac{R}{\lambda },$$ (5)

*ρ* being the Earth’s averaged density. In this experiment, both densities are close to each other *ρ* ≈ *ρ*_m_, therefore their ratio *ρ*/*ρ*_m_ is close to 1. However an adequate choice of the mirror material (coating) would easily allow one to gain a factor of three to five in the sensitivity in future experiments.

One obtains the following numerical boundary:

$$\alpha_{G}=1\times 10^{15}{\left(\frac{1\;\mu m}{\lambda }\right)}^{2}.$$ (6)

Here, 1 µm is chosen as a natural scale for this experiment.

**Fig. 1:**
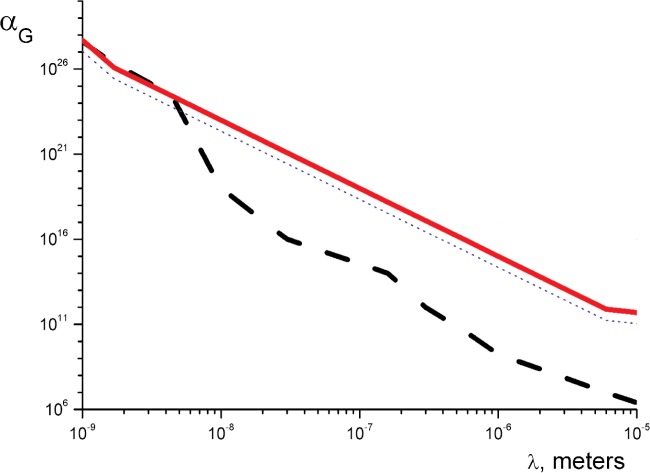
The constraints on *α_G_* following from the experiment [7] (the solid line) in comparison with that from the measurement of the Casimir and the van der Waals forces [4] (the dashed line). The dotted line shows a limit which can be easily obtained by an improvement of this experiment.

This limit is presented in Fig. 1 in comparison with the limits coming from the experiments [4]. The range of presented *λ* is 1 nm to 10 µm. A deviation from a straight line in the solid curve at 1 nm is due to the finite range of increase of the mirror effective nuclear potential (impurities on the surface and its roughness). The same effect at *λ* ≈ 10 µm is due to an “interference” of the potentials [Eqs. (2) and (3)].

## 3. Repulsive Interaction

Unfortunately, this experiment does not allow us to establish a competitive limit for a repulsive interaction. In this case, there could be no “additional” bound state. If in this experiment it would be possible to establish an experimental upper limit on the energy shift Δ*E*_n_ it would impose an upper limit on *α_G_* for a repulsive interaction [9]:

$$\frac{U_{0}m\lambda^{2}}{h^{2}}<\exp\left(\frac{\lambda_{0}}{\lambda }\right)$$ (7)

with *λ*_0_ = Δ*E*_n_/*mg*, or

$$\alpha_{G}=\frac{1}{ð}\frac{\rho }{\rho_{m}}\frac{h}{mg\lambda^{2}}\frac{h}{m\lambda }\frac{R}{\lambda }\exp\left(\frac{\lambda_{0}}{\lambda }\right).$$ (8)

One can see that the limit [Eq. (8)] at small *λ* is sufficiently less restrictive than that for an attractive one [Eq. (6)] due to the exponential factor.

## 4. Occupation Numbers

The considerations presented above are valid only if the neutron population in the lowest quantum state in such a system (with an additional interaction included) is sufficiently high to provide a measurable signal/noise ratio. The experiment [7] would allow one to identify an additional quantum state if its occupation number would not be suppressed by more than a factor of 200 compared to that for other states. In order to calculate the occupation numbers, let us start with a general expression for the probability of a rapid transition from a state *k* with the wave function *Ψ_k_*(*x*) to a state *n* with the wave function *Φ_n_*(*x*) which is given by:

$$w_{k\to n}={\left|\int \Psi_{k}\;(x)\;\Phi_{n}\;(x)\;dx\right|}^{2}.$$ (9)

for a few initial quantum states, the probability *w_n_* is a sum (an integral for continius spectrum) over them:

$$w_{n}=\sum_{n}f_{k}w_{k\to n}.$$ (10)

with the occupation numbers *f_k_* of initial states.

To obtain an analytical expression for the occupation numbers, let us consider a simplified model of a harmonic oscillator in a final state and a plane wave in an initial one. An explicit analytical shape of the final state wave function does not play a role (the only important parameter is its spatial size *x*_0_) and would not modify considerably the occupation numbers.

If initial states are populated according to the Gaussian law with a characteristic momentum *k*_0_ then and all integrals [Eq. (9)] can be calculated analytically. For instance, for the lowest states with *n* = 0 and *n* = 1:

$$w_{0}=\frac{k_{0}x_{0}}{\sqrt{1+{\left(k_{0}x_{0}\right)}^{2}}};\;w_{1}=w_{0}^{3}.$$ (11)

If *k*_0_*x*_0_ » 1 then the occupation numbers are approximately equal for all states: *w_n_* ≈ 1.

For the gravitational quantum states, *x*_0_ ≈ 6 µm; the vertical velocity distribution has a characteristic velocity of *v*_0_ ≈ 50 cm/s. For these states, *k*_0_*x*_0_ ≈ 50 » 1 and all states should have approximately the same occupation numbers.

If an additional bound state were created by the interaction [Eq. (3)] then the characteristic size of such a state should be of the order of *λ* (or bigger). For the interaction range, for which this experiment establishes a competitive limit, one obtains *w* ≈ *k*_0_*λ* ≈ 0.1 for *λ* = 10 nm and *w* ≈ *k*_0_*λ* ≈ 0.01 for *λ* = 1 nm. If such a state exists it would be detected in this experiment.

## 5. Conclusions

An upper limit to an additional attractive force is established from the measurement of quantum states of neutrons in the Earth’s gravitational field. Relatively high sensitivity of the experiment [7] to a hypothetical additional force is due to the following factors: firstly, no “background’” electromagnetic interactions; secondly, the characteristic size of the neutron wave function in the quantum states fits well to the range of interest for the short-range forces; finally, non-negligible probability to find neutrons (quantum-mechanical object) at distances much closer to the mirror than the average value of 10 µm.

The limit [Eq. (6)] improves the existing constraints [4] in the nanometer range even if this experiment was neither conceived nor optimized to establish this limit. However, it can be easily improved in the same kind of experiment with some evident modifications, for instance, one can choose a mirror material (coating) with higher density.

## References

[1] Murayama H, Raffelt GG, Hagmann C, van Bibber K, Rosenberg LJ (2002). Review of Particle Physics. Phys Rev.

[2] Arkani-Hamed N, Dimopoulos S, Dvali G (1998). Phys Lett.

[3] Hewett J, March-Russell J (2002). Review of Particle Physics. Phys Rev.

[4] Bordag M, Mohideen U, Mostepanenko VM (2001). Phys Rep.

[5] Frank A, van Isaker P, Gomes-Camacho J (2004). Phys Lett.

[6] Bertolami O, Nunes FM (2003). Class Quantum Grav.

[7] Nesvizhevsky VV, Börner HG, Gagarski AM, Petukhov AK, Petrov GA, Abele H, Baessler S, Divkovic G, Ruess FJ, Stöferle T, Westphal A, Strelkov AV, Protasov KV, Voronin AYu (2003). Phys Rev.

[8] Abele H, Westphal A (2002). ILL Annual Report.

[9] Nesvizhevsky VV, Protasov KV (2004). hep-ph/0401179.

